# The William F. Jenks Memorial Prize

**Published:** 1894-06

**Authors:** 


### The William F. Jenks Memorial Prize.

The Third Triennial Prize, of Five Hundred Dollars, under the Deed of Trust of Mrs. William F. Jenks, will be awarded to the author of the best essay on "Infant Mortality During Labour, and its Prevention." The prize is open for competition to the whole world; but the essay must be the production of a single person. The essay, which must be written in the English language, or if in a foreign language, accompanied by an English translation, should be sent to the College of Physicians of Philadelphia, Pennsylvania, U.S.A., before January 1st, 1895, addressed to Horace Y. Evans, M.D., Chairman of the William F. Jenks Prize Committee. Each essay must be typewritten, distinguished by a motto, and accompanied by a sealed envelope bearing the same motto, and containing the name and address of the writer. No envelope will be opened except that which accompanies the successful essay. Application for any further information should be made to James V. Ingham, the Secretary of the Trustees.

